# A Review of Magnetic Flux Leakage Nondestructive Testing

**DOI:** 10.3390/ma15207362

**Published:** 2022-10-20

**Authors:** Bo Feng, Jianbo Wu, Hongming Tu, Jian Tang, Yihua Kang

**Affiliations:** 1 School of Mechanical Science and Engineering, Huazhong University of Science and Technology, Wuhan 430074, China; 2 School of Mechanical Engineering, Sichuan University, Chengdu 610065, China

**Keywords:** MFL, NDT, sensor, magnetic dipole model, high speed, liftoff, magnetizer, inverse problem, artificial neural network

## Abstract

Magnetic flux leakage (MFL) testing is a widely used nondestructive testing (NDT) method for the inspection of ferromagnetic materials. This review paper presents the basic principles of MFL testing and summarizes the recent advances in MFL. An analytical expression for the leakage magnetic field based on the 3D magnetic dipole model is provided. Based on the model, the effects of defect size, defect orientation, and liftoff distance have been analyzed. Other influencing factors, such as magnetization strength, testing speed, surface roughness, and stress, have also been introduced. As the most important steps of MFL, the excitation method (a permanent magnet, DC, AC, pulsed) and sensing methods (Hall element, GMR, TMR, etc.), have been introduced in detail. Finally, the algorithms for the quantification of defects and the applications of MFL have been introduced.

## 1. Introduction

Magnetic flux leakage (MFL) testing is an electromagnetic nondestructive testing (NDT) method with high efficiency and reliability. It has the ability to detect various types of defects such as cracks, corrosion, pitting, and cavity, and it is able to detect both surface and subsurface defects. Therefore, it has been widely used to ensure the integrity and safety of structures in the petrochemical, energy, manufacturing, and transportation industries. 

The principle of MFL testing is based on the interaction between magnetic field and defects. The MFL testing device usually consists of a magnetizing unit, a sensing unit, a signal conditioning unit, an analog-to-digital converter (ADC), and a computer with signal displaying and analyzing software. The magnetizing unit is usually consisting of permanent magnets of magnetizing coils that are able to magnetize the ferromagnetic specimen into saturation or near saturation. Due to the abrupt change in magnetic reluctance at the defects, the magnetic flux leaks into the nearby air. The perturbation of the magnetic field can be recorded by an array of magnetic field sensors and used to evaluate and quantify defects.

MFL testing theory and technology have been developed for decades. There are several review papers that summarized some developments in MFL together with other electromagnetic NDT methods [1,2,3,4]. However, there is no comprehensive review of MFL technology. In the following sections of this paper, a comprehensive review will be given to the following subjects of MFL technology: (1) the study of the MFL principle and analytical model; (2) the influence of testing parameters (e.g., magnetizing strength, liftoff, scanning speed) and defect properties (e.g., defect size and defect orientation) on the MFL signals; (3) excitation and sensing techniques in MFL testing; (4) inverse problem and defect quantification in MFL; (5) applications of MFL and comparison with related NDT methods. 

## 2. MFL Principle and Analytical Model

### 2.1. MFL Principle

**Figure 1:**
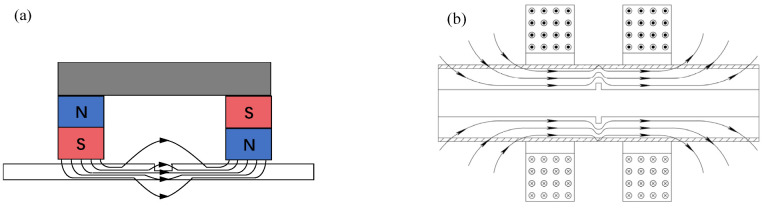
Basic principle of MFL testing: (**a**) yoke-type magnetizer; (**b**) encircling coil-type magnetizer.

The basic principle of MFL testing is schematically shown in Figure 1, where a magnetizer is applied to magnetize the ferromagnetic specimen into near saturation. The magnetizer can be either a magnet with a ferromagnetic yoke or a magnetizing coil. Due to the high permeability of ferromagnetic materials, the magnetic flux is constrained in the material when no defects are presented. In the presence of a defect, the magnetic field leaks into the nearby air and causes the leakage field.

**Figure 2:**
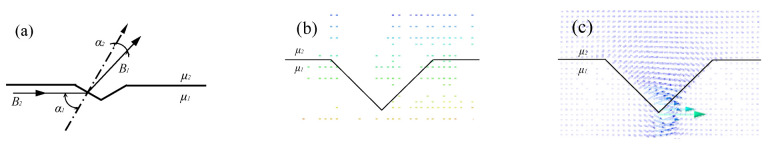
The refraction of magnetic field at interface of a defect: (**a**) schematic representation; (**b**) finite element simulation results of magnetic vectors for *μ*_1_ = *μ*_2_; (**c**) finite element simulation results of magnetic vectors for *μ*_1_ > *μ*_2_.

The phenomenon of magnetic field leakage was explained using the refraction of the magnetic field by Sun and Kang with the boundary conditions of the electromagnetic field [5], as shown in Figure 2a. At the interface of the two media, the magnetic fields satisfy the boundary equations; thus, the refracted angle is expressed as:

(1) $$\alpha_{2}=\arctan(\frac{\mu_{2}}{\mu_{1}}\tan\alpha_{1})$$

where *μ*_1_ and *μ*_2_ are the permeabilities of medium 1 and 2, respectively.

Therefore, if *μ*_1_ = *μ*_2_, which is the case where the specimen is non-ferromagnetic, then *α*_1_ = *α*_2_. In this case, the flux line continues at the interface without any perturbation, and there is no leakage magnetic field. If *μ*_1_ > *μ*_2_, then *α*_1_ > *α*_2_, and the magnetic field will enter the vicinity of the defect due to refraction.

### 2.2. Forward Problem and Magnetic Dipole Model 

**Figure 3:**
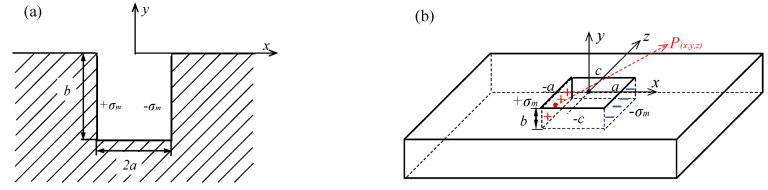
Dipole model for the magnetic field calculation: (**a**) 2D representation; (**b**) 3D representation.

The forward problem, which derives the MFL field of a defect with a certain shape, is a fundamental and important topic in MFL testing. One of the most commonly used models in the forward problem is the magnetic dipole model, the study of which was pioneered by Zatsepin and Shcherbinin [6,7]. Based on their study, many researchers have further derived the distribution of the leakage field generated by a 2D notch and a 3D notch [8,9]. The dipole is assumed to distribute uniformly at the slot surfaces with the density *σ*_m_. For a line with infinitesimal length d*y* on the slot surface shown in Figure 3a, the magnetic charge is $dp=\sigma_{m}dy$, and in the 3D model shown in Figure 3b, the charge is $dp=\sigma_{m}dydz$. The magnetic field generated by the magnetic charge is $dH=\frac{dp}{4\pi r^{3}}r$. By taking the integral for all magnetic charges at slot surfaces, the magnetic field can be derived. For the 2D model in Figure 3a, the tangential and normal components of the field are:

(2) $$H_{x}(x,y)=\frac{\sigma_{m}}{2\pi }\left[\arctan\frac{b(x+a)}{{\left(x+a\right)}^{2}+y(y+b)}-\arctan\frac{b(x-a)}{{\left(x-a\right)}^{2}+y(y+b)}\right]$$

(3) $$H_{y}(x,y)=\frac{\sigma_{m}}{4\pi }\ln\left(\frac{\left[{\left(x+a\right)}^{2}+{\left(y+b\right)}^{2}][{\left(x-a\right)}^{2}+y^{2}\right]}{\left[{\left(x+a\right)}^{2}+y^{2}][{\left(x-a\right)}^{2}+{\left(y+b\right)}^{2}\right]}\right)$$

For the 3D model in Figure 3b, the results are:

(4) $$\begin{array}{ll} H_{x}\left(x,y,z\right) & =\frac{\sigma_{m}}{4\pi }(\arctan\frac{\left(y+b\right)\left(z+c\right)}{\left(x+a\right){\left[{\left(x+a\right)}^{2}+{\left(y+b\right)}^{2}+{\left(z+c\right)}^{2}\right]}^{1/2}}-\arctan\frac{y\left(z+c\right)}{\left(x+a\right){\left[{\left(x+a\right)}^{2}+y^{2}+{\left(z+c\right)}^{2}\right]}^{1/2}}\\ & -\arctan\frac{\left(y+b\right)\left(z-c\right)}{\left(x+a\right){\left[{\left(x+a\right)}^{2}+{\left(y+b\right)}^{2}+{\left(z-c\right)}^{2}\right]}^{1/2}}+\arctan\frac{y\left(z-c\right)}{\left(x+a\right){\left[{\left(x+a\right)}^{2}+y^{2}+{\left(z-c\right)}^{2}\right]}^{1/2}}\\ & -\arctan\frac{\left(y+b\right)\left(z+c\right)}{\left(x-a\right){\left[{\left(x-a\right)}^{2}+{\left(y+b\right)}^{2}+{\left(z+c\right)}^{2}\right]}^{1/2}}+\arctan\frac{y\left(z+c\right)}{\left(x-a\right){\left[{\left(x-a\right)}^{2}+y^{2}+{\left(z+c\right)}^{2}\right]}^{1/2}}\\ & +\arctan\frac{y\left(z-c\right)}{\left(x-a\right){\left[{\left(x-a\right)}^{2}+{\left(y+b\right)}^{2}+{\left(z-c\right)}^{2}\right]}^{1/2}}-\arctan\frac{y-a)}{\left(x-a\right){\left[{\left(x-a\right)}^{2}+y^{2}+{\left(z-c\right)}^{2}\right]}^{1/2}}) \end{array}$$

(5) $$\begin{array}{ll} H_{y}\left(x,y,z\right) & =\frac{\sigma_{m}}{4\pi }[\ln(\frac{z+c+{\left[{\left(x+a\right)}^{2}+y^{2}+{\left(z+c\right)}^{2}\right]}^{1/2}}{z-c+{\left[{\left(x+a\right)}^{2}+y^{2}+{\left(z-c\right)}^{2}\right]}^{1/2}}\times \frac{z-c+{\left[{\left(x+a\right)}^{2}+{\left(y+b\right)}^{2}+{\left(z-c\right)}^{2}\right]}^{1/2}}{z+c+{\left[{\left(x+a\right)}^{2}+{\left(y+b\right)}^{2}+{\left(z+c\right)}^{2}\right]}^{1/2}})\\ & -\ln(\frac{z+c+{\left[{\left(x-a\right)}^{2}+y^{2}+{\left(z+c\right)}^{2}\right]}^{1/2}}{z-c+{\left[{\left(x-a\right)}^{2}+y^{2}+{\left(z-c\right)}^{2}\right]}^{1/2}}\times \frac{z-c+{\left[{\left(x-a\right)}^{2}+{\left(y+b\right)}^{2}+{\left(z-c\right)}^{2}\right]}^{1/2}}{z+c+{\left[{\left(x-a\right)}^{2}+{\left(y+b\right)}^{2}+{\left(z+c\right)}^{2}\right]}^{1/2}})] \end{array}$$

(6) $$\begin{array}{cc} H_{z}(x,y,z) & =\frac{\sigma_{m}}{4\pi }[\ln(\frac{y+b+{\left[{\left(x+a\right)}^{2}+{\left(y+b\right)}^{2}+{\left(z-c\right)}^{2}\right]}^{1/2}}{y+{\left[{\left(x+a\right)}^{2}+y^{2}+{\left(z-c\right)}^{2}\right]}^{1/2}}\times \frac{y+{\left[{\left(x+a\right)}^{2}+y^{2}+{\left(z+c\right)}^{2}\right]}^{1/2}}{y+b+{\left[{\left(x+a\right)}^{2}+{\left(y+b\right)}^{2}+{\left(z+c\right)}^{2}\right]}^{1/2}})\\ & -\ln(\frac{y+b+{\left[{\left(x-a\right)}^{2}+{\left(y+b\right)}^{2}+{\left(z-c\right)}^{2}\right]}^{1/2}}{y+{\left[{\left(x-a\right)}^{2}+y^{2}+{\left(z-c\right)}^{2}\right]}^{1/2}}\times \frac{y+{\left[{\left(x-a\right)}^{2}+y^{2}+{\left(z+c\right)}^{2}\right]}^{1/2}}{y+b+{\left[{\left(x-a\right)}^{2}+{\left(y+b\right)}^{2}+{\left(z+c\right)}^{2}\right]}^{1/2}})] \end{array}$$

**Figure 4:**
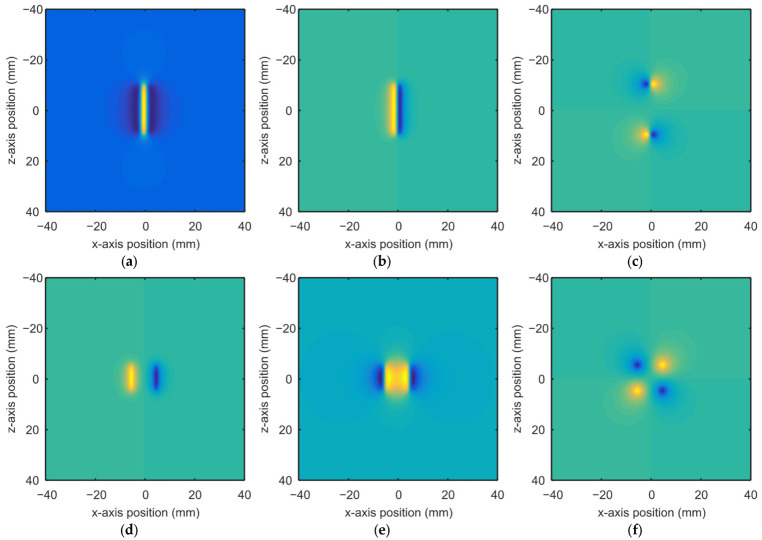
Distribution of magnetic field above defects with colors indicating the intensity of magnetic field: (**a**) *H_x_* for a rectangular notch; (**b**) *H_y_* for a rectangular notch; (**c**) *H_z_* for a rectangular notch; (**d**) *H_x_* for a square notch; (**e**) *H_y_* for a square notch; (**f**) *H_z_* for a square notch.

According to Equations (4)–(6), the distribution of magnetic field above a notch can be calculated. Figure 4 shows the magnetic field distributions at liftoff *y* = 1 for defects with the dimensions of *a* = 1, *b* = 2, *c* = 10 and *a* = 5, *b* = 2, *c* = 5.

The magnetic dipole model was further extended to calculate the magnetic field generated by defects with various shapes. Uetake studied the MFL of adjacent parallel surface slots [10]. Dutta and Stanley calculated the MFL of a cylindrical hole and verified the model by comparing it with simulation results [11,12]. Mandache and Clapham calculated the MFL of a cylindrical hole, a racetrack defect, and adjacent holes [13]. Lukyanets derived an analytical model for the MFL of a defect with a smooth surface [14]. Trevino proposed an improved dipole model to calculate the MFL of conical, ellipsoidal, and tensional shaped defects [15]. Wu proposed a model for concave and bump shaped defects [16]. Zhang derived an analytical expression for internal defects using a modified dipole model and image theory [17]. Li proposed to improve the accuracy of the dipole model by considering a two-layer charge distribution model [18].

In addition to the commonly used magnetic dipole model, there are also some analytical models derived for the calculation of the MFL. Bowler derived an analytical solution for semi-elliptical indentation by solving the Laplace equation of a static magnetic field, and the results turned to be in agreement with those from the dipole model [19]. Cheng and Wang proposed a solenoid model based on the magnetization mechanisms of the magnetic medium, and calculated the V-shaped and Z-shaped defects [20,21]. Huang used a basic signal analysis approach to predict the MFL response with high accuracy and calculation speed [22].

## 3. Factors Influencing MFL Signal

In MFL testing, there are many factors that influence the inspection signal. This section summarizes some of the important factors such as the defect size, defect orientation, liftoff distance, magnetization strength, stress, and scanning velocity.

### 3.1. Defect Dimension and Orientation

The influence of defect size on the MFL signal has been analyzed using analytical models, simulations, and experiments [8,23,24,25,26,27,28,29]. It was suggested by Förster that [8], when using the magnetic dipole model to analyze the influence of defect dimension, the magnetic charge density should also change with defect dimension to obtain more accurate results:

(7) $$\sigma_{m}=\frac{1}{2\pi }\frac{b/a+1}{(1/\mu )b/a+1}F_{\mathrm{nl}}H_{a}$$

where *F*_nl_ is a non-linear factor and *H*_a_ is the applied field.

**Figure 5:**
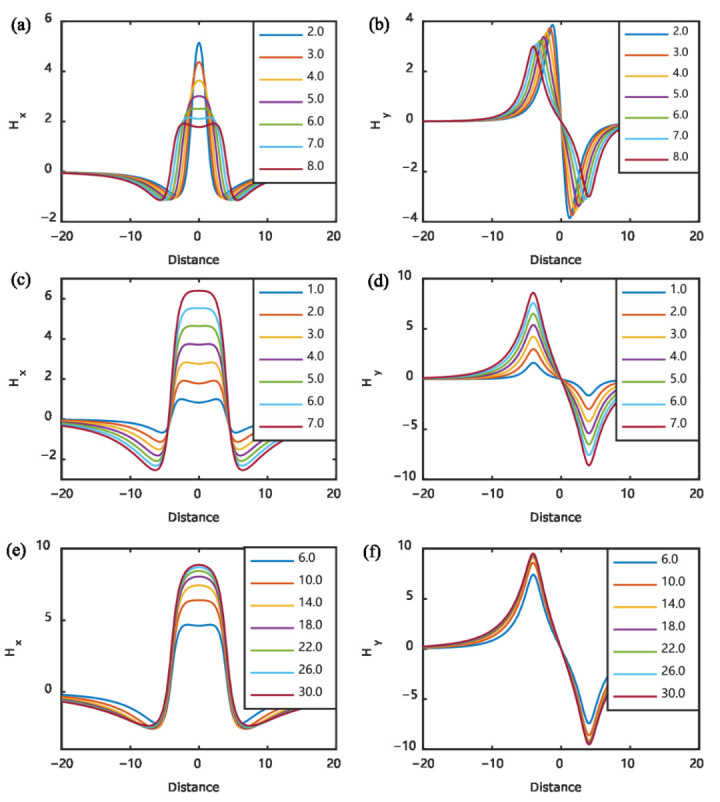
Influences of defect dimensions on MFL signal: (**a**) change in *H_x_* with defect width; (**b**) change in *H_y_* with defect width; (**c**) change in *H_x_* with defect depth; (**d**) change in *H_y_* with defect depth; (**e**) change in *H_x_* with defect length; (**f**) change in *H_y_* with defect length.

The influence of the defect dimensions can be analyzed using Equations (4)–(7). For the defect shown in Figure 3b, the magnetic field above the center of the defect (*y* = 1, *z* = 0) was extracted and the results are shown in Figure 5.

**Figure 6:**
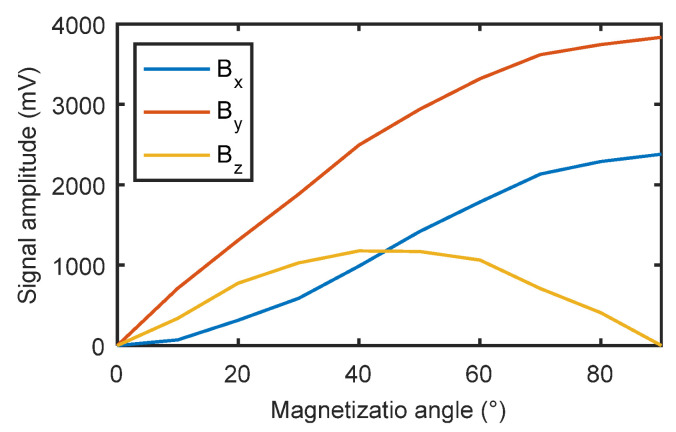
Influence of the angle between magnetization and defect on MFL signal.

Conventionally, researchers and engineers of MFL have thought that the orientation defect should be perpendicular to the magnetization field to obtain an effective MFL field. Sun and Song questioned this traditional conclusion and studied the MFL signals for defects parallel to the magnetization field [30,31]. They found that it was possible to detect cracks that are parallel to the magnetization, although the amplitude was small. Wu further studied the variation of MFL signal amplitude with the angle between the defect and magnetization field [32], the results are shown in Figure 6. 

**Figure 7:**
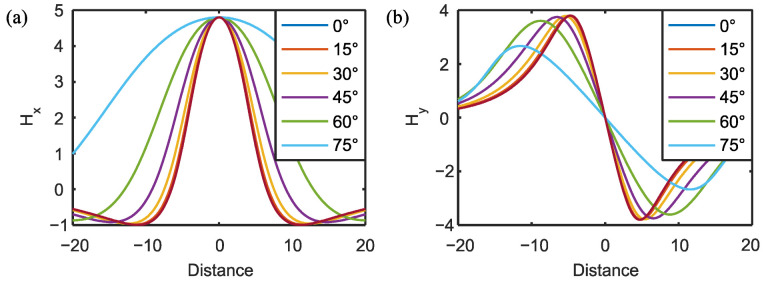
Influence of scanning angle on MFL signal: (**a**) change in *H_x_* with scanning angle; (**b**) change in *H_y_* with scanning angle.

The scanning direction of the sensors also influences the MFL signal, and Wu also studied this effect [32]. This effect can also be obtained by extracting the magnetic field along different directions using Equations (4)–(6). For a defect with the dimensions *a* = 1, *b* = 2, and *c* = 10, the variation of the MFL signal with scanning direction is shown in Figure 7.

### 3.2. Liftoff Effect

**Figure 8:**
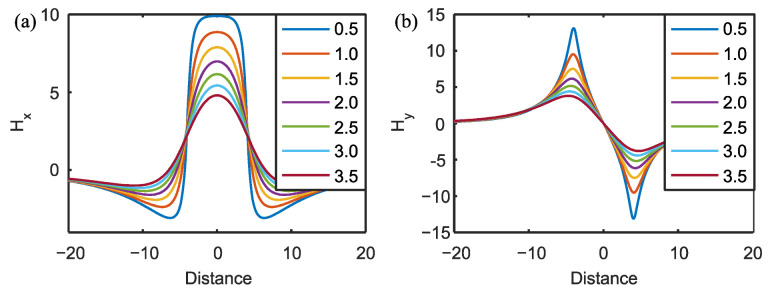
Influence of liftoff on MFL signal: (**a**) change in *H_x_* with liftoff; (**b**) change in *H_y_* with liftoff.

The MFL signal is dependent on the liftoff distance between the probe and the specimen. The MFL signal reduces as the increase in liftoff distance [33,34,35], an example of liftoff effect is shown in Figure 8. 

The change in liftoff during scanning significantly influences the testing signal. Thus, many researchers have attempted to reduce the liftoff effect. Jia used a filtering method to suppress liftoff interference [36]; Wu proposed a liftoff tolerant sensor by inserting ferrite into the sensing coil [37]; Peng introduced an exponential function compensation for liftoff correction [38]; Wang linearized the liftoff effect by applying Fourier transform [39].

### 3.3. Magnetization Strength and Material Property

**Figure 9:**
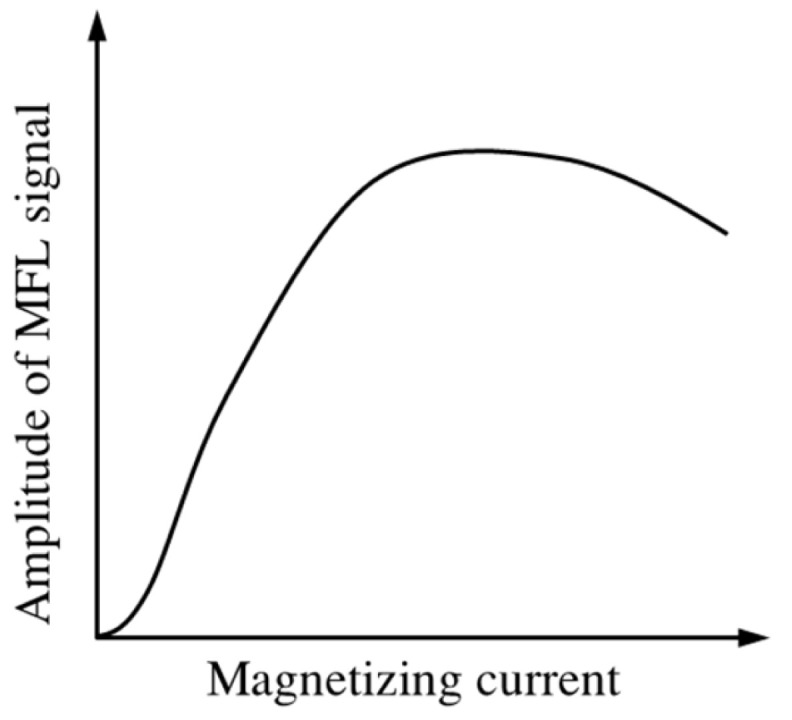
Influence of magnetizing current on MFL signal.

Usually, a strong magnetization is required to saturate the ferromagnetic material to obtain a good MFL signal. However, the MFL signal does not always increase with magnetization strength. Many researchers have found that the MFL signal initially increases with the magnetizing current and starts to decrease after a certain point [40,41,42], as shown in Figure 9. Sun explained this phenomenon with the magnetic compression effect [43], which states that the large background field caused by strong magnetization suppresses the leakage of the magnetic field from defects. Later, he proposed a new MFL principle based on near-zero background magnetic field [44], in which magnetic shielding is used to collect a strong background field.

Since the magnetization of the material is also based on the material property, the MFL signal is also dependent on the B–H curve of the ferromagnetic materials. Katoh approximated the B–H curve with two lines and studied this influence [45].

### 3.4. Velocity Effect

In pipeline inspection, the MFL device is propelled by the gas and oil inside the pipe. The device usually travels several meters per second. Due to the relatively motion between the magnetizer and the pipe, eddy currents are induced in the pipe wall. The motion-induced eddy current density is:

(8) J=σv×B

The eddy currents generate a secondary magnetic field according to the Biot–Savart law. Thus, the magnetization status of the pipe and corresponding MFL signal will be affected at high testing speeds. The problem is governed by Maxwell’s equations considering the velocity term:

(9) $$\nabla^{2}B-\mu \sigma \frac{\partial B}{\partial t}-\mu \sigma \left(v\cdot \nabla \right)B=0$$

**Figure 10:**
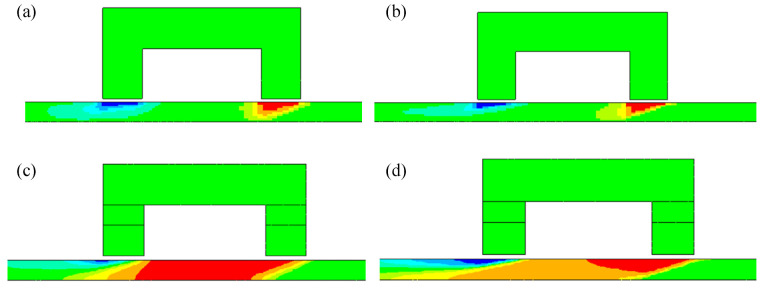
Distribution of motion-induced eddy current and magnetic field: (**a**) eddy current at 0.5 m/s; (**b**) eddy current at 2 m/s; (**c**) magnetic field at 0.5 m/s; (**d**) magnetic field at 2 m/s.

Many researchers have studied the velocity effect of finite element simulation. For the yoke-type magnetizer, the eddy currents are induced in the region beneath the poles [46,47,48,49], and the magnetic field is perturbed [50]. A comparison between the distributions of the eddy current and magnetic field at different speeds is shown in Figure 10. 

Recently, B. Feng found an analytical solution to Equation (9) and further obtained the expression of the motion-induced eddy current under a pole of the magnetizer [51]:

(10) $$J_{\mathrm{total}}=\frac{1}{\pi }\cdot \int_{0}^{\infty }dk\int_{y_{0}}^{y_{0}+h}[\frac{1}{\mu }(jkC_{y}^{\mathrm{III}}e^{k\prime y}+jkD_{y}^{\mathrm{III}}e^{-k\prime y}-k\prime C_{x}^{\mathrm{III}}e^{k\prime y}+k\prime D_{x}^{\mathrm{III}}e^{-k\prime y})]e^{jkx}dy\prime$$

where *J*_total_ is the eddy current in specimen, *y*_0_ is the liftoff, *h* is the height of magnetizer, $C_{x}^{\mathrm{III}}$, $C_{y}^{\mathrm{III}}$, $D_{x}^{\mathrm{III}}$ and $D_{y}^{\mathrm{III}}$ are coefficients that are solved as [51]:

$$C_{x}^{\mathrm{III}}=-\frac{2j\mu Ik\prime \left(k\prime +k\right)e^{-ky\prime +k\prime d}\sin\left(ka\right)}{e^{k\prime d}{\left(k\prime +k\right)}^{2}-e^{-k\prime d}{\left(k\prime -k\right)}^{2}}$$

$$D_{x}^{\mathrm{III}}=\frac{2j\mu Ik\prime \left(k\prime -k\right)e^{-ky\prime -k\prime d}\sin\left(ka\right)}{e^{k\prime d}{\left(k\prime +k\right)}^{2}-e^{-k\prime d}{\left(k\prime -k\right)}^{2}}$$

$$C_{y}^{\mathrm{III}}=-\frac{2\mu Ik\left(k\prime +k\right)e^{-ky\prime +k\prime d}\sin\left(ka\right)}{e^{k\prime d}{\left(k\prime +k\right)}^{2}-e^{-k\prime d}{\left(k\prime -k\right)}^{2}}$$

$$D_{y}^{\mathrm{III}}=-\frac{2\mu Ik\left(k\prime -k\right)e^{-ky\prime -k\prime d}\sin\left(ka\right)}{e^{k\prime d}{\left(k\prime +k\right)}^{2}-e^{-k\prime d}{\left(k\prime -k\right)}^{2}}$$

With the analytical solution of the MIEC, the tail effect and tilt angle of the MIEC at different moving speeds are also analyzed [51].

The encircling coil-type magnetizer is more commonly used in the manufacturing line for the inspection of steel pipes. As the production speed increases, there is also a need for high-speed testing. For the encircling coil-type magnetizer, the motion-induced eddy currents are mainly focused on the edge of the magnetizing coil [52,53,54,55,56]. Wu also studied the distribution of eddy current in circumferential-type MFL testing [57]. 

The influence of the velocity on the MFL signal has also been extensively studied. There are changes in both the signal baseline and signal amplitude, and the signal shape is also distorted [58,59,60,61]. The change in MFL signal amplitude with velocity has been reported in many previous studies, some have reported that the signal amplitude decreases with the increase in velocity [48,52]. However, further detailed analyzes by Pullen showed that when there is insufficient flux saturating the specimen, the MFL signal for far-side defects decreases with scanning speed, while the signal for near-side defects increases with the speed [62,63]. Zhang further found that the influence of the velocity also depends on the sensor position [64]. In order to reduce the velocity impact, Usarek studied the change in the magnetic field with velocity and found that both tangential and normal components of the magnetic field increase with velocity linearly and used an empirical fitting equation to compensate for the MFL signal [65]. 

Based on the studies of the velocity effect in MFL testing, many researchers have attempted to use the velocity-induced field for testing. Antipov used an induced tail magnetic field to test rails at high-speed [66]. B. Feng, T. Rocha, and F. Yuan all studied the motion-induced eddy current testing method and used magnetic field sensors to pick up the defect signals [51,67,68,69,70,71,72]. Researchers from Technische Universität Ilmenau proposed a new Lorentz force NDT method for conductive specimens, which is also based on motion-induced eddy current [73,74,75,76,77].

### 3.5. Other Effects

In MFL testing, there are also other factors that influence the MFL signal, such as stress, surface roughness, corrosion coverage, and probe gesture. Kasai studied the MFL testing signal for samples covered by corrosion (iron oxides) and showed that the MFL signal decreased with increasing iron oxide ratio [78]. Long studied the influence of gesture probes on MFL signal and proposed a dual magnetic sensor model to compensate for the change in probe gesture [79].

The stress effect has been studied by many researchers [80,81,82,83,84,85,86,87,88,89,90,91]. The properties of ferromagnetic materials change with the loading stress due to the magneto-mechanical coupling, thus the MFL signal also changes with the stress. Y. Wang proposed a multi-physics simulation model to study the change in MFL signal with stress and showed that the peak-to-peak amplitude of the normalized MFL signal decreases with an increase in stress [80]. Mandal showed that the circumferential bending stress changes the magnetic easy axis of the pipe and thus reduces the MFL signal [81]. Y. Wang also studied the stress-dependent MFL signals in Q235 steel plates [82]. Timoshenko’s theory and the J-A model were combined to calculate the stress-dependent distribution of magnetization, then a modified magnetic dipole model considering the stress dependence was proposed. With the proposed model, Wang showed that the MFL signal increases with the increase in stress in the Q235 steel plate. Gao also observed a similar effect in the testing of steel wire ropes [83]. Later, Shi showed that the change in the MFL signal behaves differently in the elastic and plastic deformation stage [84].

For the testing of micro-cracks, the influences of surface roughness cannot be ignored. Deng considered the rough surface as concave and convex defects and showed that a rough surface introduces background noise to the MFL signal and reduces the signal-to-noise ratio (SNR) [92]. Yang also studied the effect of surface roughness on the SNR of MFL signals and proposed the use of a magnetic medium to improve the SNR [93]. In another study, B.P.C. Rao proposed the use of an Eigen vector-based approach to suppress the noise caused by non-linear permeability, surface roughness, stresses, and liftoff variations in MFL images [94]. Since the surface roughness influences the MFL signal, the MFL signal can be used in turn to represent the surface roughness. Li proposed to use the MFL signal and its spatial Fourier spectrum to measure surface roughness [95].

## 4. Excitation and Sensing Techniques in MFL Testing

### 4.1. Excitation Methods

#### 4.1.1. Structures of Magnetizer

In conventional MFL testing, the excitation field is provided by either permanent magnets or direct current (DC) carrying coils. The main advantages of using permanent magnets include: (1) the magnetizer has a relatively small size and light weight; (2) there is no need for an external power supply. Due to these features, magnet-based magnetizers are especially suitable for use in portable devices and inspection robots for the inspection of wire ropes and transmission pipelines. The drawback of using permanent magnets is that the installation is not convenient due to the large magnetic force between the magnet and the specimen, and the magnetization strength is difficult to adjust. These drawbacks can be overcome using coils. The magnetization strength can be easily adjusted by changing the current in the coil and the current can be turned off during the installation. The distribution of the magnetic field generated by magnetizing coils can be calculated through magnetic vector potentials [96], and a uniform magnetizing field can be achieved by the design of Helmholtz coils [97]. However, coil-based magnetizers usually have larger sizes; thus, they have limitations in some applications. With either a permanent magnet or coil, a ferromagnetic yoke can be used to formulate magnetic circuits with less magnetic reluctance to increase the magnetization inside the specimens.

**Figure 11:**
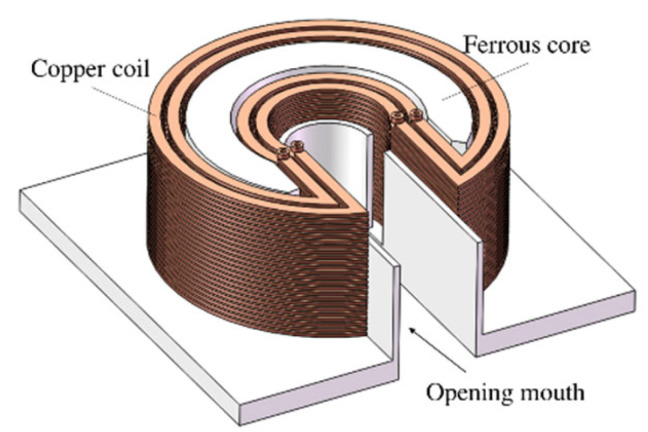
The structure of the opening electromagnetic transducer.

The encircling coil-based magnetizer has the advantage of providing a strong and adjustable magnetizing field; however, wires are closed, making it difficult for certain specimens such as wire ropes and coiled tubing to be inserted into the middle of the coil. To solve this problem, Y. Sun proposed an opening electromagnetic transducer, as shown in Figure 11, which facilitates the insertion of specimens [98,99]. S. Wang proposed a flexible magnetizer-based parallel cable that may have potential applications in specimens with complex curvature [100].

**Figure 12:**
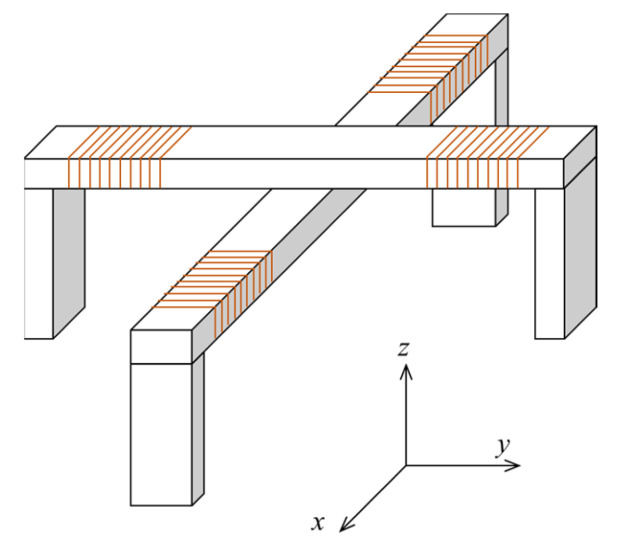
The structure of double U-shaped orthogonal magnetizer.

For the yoke type magnetizer, Y. Chang did several optimizations for the yoke shape, yoke size and thickness of shielding layer with the help of finite element simulation [101]. J. Parra-Raad performed a multi-object optimization for pipeline inspection gauge (PIG) by the genetic algorithm [102]. The conventional yoke type magnetizer only generates a magnetic field in one direction and has limited sensitivity for cracks that are parallel to the magnetic field. A double U-shaped orthogonal magnetizer, as shown in Figure 12, can be used to overcome this problem, although it was originally developed for alternating current field measurement (ACFM) [103]. When AC excitation is used in the MFL testing, the direction of the magnetizing field can be adjusted by controlling the phase difference between the two yokes.

#### 4.1.2. Excitation Signal Waveforms

To extract more defect information in MFL testing, researchers have considered the optimization of the excitation signal waveform. Alternating current magnetic flux leakage (ACMFL), pulsed magnetic flux leakage (PMFL), and MFL-based combined AC and DC excitation have been proposed. Y. Gotoh conducted a comprehensive study on ACMFL, analyzed ACMFL with finite element simulation, stated the necessity of using nonlinear analysis, and used the method to detect plural cracks [104,105]. Due to the skin effect, the magnetic field concentrates on the surface of the specimen, thus the surface can be saturated with a relatively small excitation current. Gotoh also used low-frequency AC excitation to increase the penetration depth and detected outer side cracks in a steel plate 3 mm thick [106,107]. Hayashi proposed an unsaturated ACMFL testing method for reinforcing steel bars and achieved defect inspection at high liftoff of up to 100 mm [108].

To increase the depth of penetration and obtain richer information, A. Sophian and G.Y. Tian proposed the PMFL method, in which a square waveform is applied as the excitation signal [109]. It was found that the PMFL method has advantages in defect location and sizing. J. Wilson combined the PMFL method with pulsed magnetic reluctance (PMR), which provided a complementary approach for the integrated inspection of surface and sub-surface cracks [110]. Subsequently, many researchers have studied the signal characteristics and extracted features for defect quantification and discrimination of internal and external defects [111,112,113,114].

The combined DC and AC excitation has also been used in MFL testing. D. Wu proposed a magnetizer with both permanent magnets and coils excited with alternating current [115]. The AC excitation was used to generate eddy currents that were perpendicular to the DC magnetic field to cover the blind zone of the DCMFL. R. Wang proposed to use two encircling coils to, respectively, generate DC and AC magnetizing fields [116], in which the DC field is used to set the working point by changing the permeability and the AC field is used to obtain the defect information. The results showed that this method can be used to increase the detectability of internal defects. Y. Gotoh also studied the combinational use of DC and AC excitation and took into account the minor hysteresis loop in the detection of far-side defects [117].

### 4.2. Sensing Methods

After generating a leakage field with appropriate excitation, sensing is the vital step to pick up the leakage field. Various types of magnetic field sensors that can convert the magnitude of a magnetic field into the corresponding voltage have been used in MFL testing. The most commonly used sensors are the Hall element and coils. Hall element is able to measure the absolute value of the magnetic field; however, when the sensor is near the poles of the magnetizer, it may operate outside the linear range. Coils have a wider measurement range; however, they only measure the rate of change in the magnetic field instead of its absolute value. In recent studies, magnetic field sensors with higher sensitivities have been used in MFL testing for the detection of tiny cracks. Kataoka and Singh used a giant magnetoresistance (GMR) line sensor and flexible GMR sensor array in MFL [118,119]. Tehranchi used a double-core giant magneto-impedance (GMI) sensor in the testing of steel plates [120]. Z. Jin used a tunnel magnetoresistance (TMR) sensor for the inspection of steel bars [121]. Kallias and Krause discussed the potential of using a superconducting quantum interference device (SQUID) in nondestructive testing [122,123].

**Figure 13:**
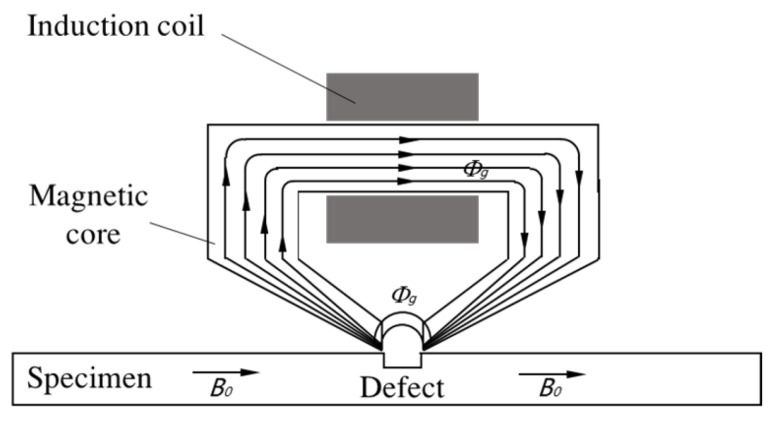
The structure of magnetic head.

In addition to using new sensing elements, researchers have also tried to enhance the MFL signal by the design of a probe structure. G. Park and Y. Jia both considered adding a ferromagnetic backing near the sensor to enhance the MFL signal [124,125]. J. Wu proposed to use a magnetic head (as shown in Figure 13) to detect tiny cracks in bearings [126], J. Tang further studied the influence of head pose on the MFL signal [127]. E. Li studied the relationship between the size of the opening in the magnetic head and the frequency of the MFL signal and designed a magnetic head structure for trans-scale defects [128,129]. S. Liu proposed a magnetic focusing sensor that adds a magnetic guide core and a permanent magnet to coils [130]. D. Wu proposed to use two sensors to measure the change rate of magnetic flux leakage to reduce background and vibration noises [131]. J. Tang proposed to use a ferromagnetic material with grooves to replace the conventional non-ferromagnetic liftoff layer to increase the MFL signal [132]. T. Nara designed a Fourier coil consisting of two coils of radial offset [133]. The sensor is able to obtain the Fourier coefficients of the leakage magnetic flux and locate the center of the crack.

Magnetic sensors are the most commonly used sensing methods in MFL testing; however, the results are not intuitive, and it requires additional signal processing circuits and display modules. J. Philip and V. Mahendran have proposed the use of a ferrofluid emulsion film to visualize the leakage magnetic field [134,135,136]. The uniformly distributed particles re-distribute under the leakage magnetic field and exhibit different colors due to Bragg scattering of the droplets. J. Lee also proposed a method to visualize the leakage magnetic field using magneto-optical film (MOF) [137]. According to the magneto-optical effect, a polarized light rotates when it is transmitted through an MOF with an external field, and the rotated angle is proportional to the external field. Thus, the MOF can be used to observe the leakage magnetic field. M. Tehranchi added a detector behind the magneto-optical sensor to capture the light and recorded the change in the magnetic field in a computer [138].

The development of the sensing method in MFL is mainly dedicated to the inspection of micro-cracks, especially the cracks in high-precision mechanical parts such as bearing and bearing roller. Researchers have optimized the sensing probe from the aspects of using highly sensitive sensors such as TMR and designing new types of structures such as magnetic heads [126,128]. In E. Li’s paper, it is reported that the smallest crack that can be detected is with a depth of 7 μm.

## 5. Inverse Problem in MFL and Defect Quantification

The ultimate goal of non-destructive testing can be classified into three levels. At the basic level, we need to determine whether there are defects in the specimen based on the testing signals. Furthermore, the defect size needs to be quantified to determine the severity of the damage. Ultimately, the defect size information will be used to predict the remaining life of the structures. After decades of development, qualitative determination of the existence of a defect is relatively simple. Thus, a lot of effort has been put into the study of the quantification of defects, which is a classical inverse problem.

### 5.1. Machine Learning-Based Defect Quantification

Machine learning has undergone rapid development in recent years, especially in the branches of artificial neural networks (ANN) and deep learning. Machine learning techniques showed great success in tasks such as classification and regression. The task of defect quantification is essentially a problem of classification or regression; thus, artificial neural networks have been widely used in defect quantification in MFL testing. The neural networks can be regarded as a function that maps the inputs (raw signal or features extracted from the signal) to the outputs (defect type, defect size, etc.). To train a neural network, experiments and simulations should be conducted for defects of various sizes. The defect sizes and corresponding signals (or signal features) are fed into the network to update the weights.

Initially, due to the limited performance of computers, shallow neural networks with one or two hidden layers were used. Carvalho used raw MFL signals and signals after filtering as the input to the neural network, and classified signals into defects and non-defects with an accuracy of 94.2% [139]. He also used the neural network to classify the defects into external corrosion, internal corrosion, and lack of penetration with an accuracy of 71.7%. K. Hwang employed the wavelet basis function (WBF) neural network to the MFL signal to a three-dimensional defect profile [140]. The WBF provides a multi-resolution approximation and overcomes some disadvantages of the radial basis function neural network. Khodayari-Rostamabad introduced various machine learning techniques and feature selection methods and estimated the defect depth with an error of less than 8% [141]. Kandroodi used the MFL signal contour to determine the defect length and width and used the signal peak-to-peak values along with the estimated length and width to estimate the defect depth [142].

With the increase in computer performance, deep neural networks that require massive computational resources have been applied in many industrial fields. The additional layers in the deep neural network can be regarded as feature extractors. Applying deep neural networks avoids manual feature extraction, a process that highly depends on the experience of the researcher and the engineer. J. Feng applied a convolutional neural network (CNN) to classify injurious and noninjurious defects based on MFL images and showed that CNN gave more accurate predictions than neural networks, support vector machines, decision trees, and correlation-based methods [143]. S. Lu proposed a visual transformation CNN for defect quantification and improved the accuracy of estimation for length, width, and depth by 26.9%, 27.1%, and 33.3% [144]. Z. Wu used reinforcement learning to replace the classic iteration process and successfully reconstructed complex defect depth profiles [145]. H. Sun stated that taking into account the physical concepts in the deep neural network would be better than only using general neural networks [146]. He integrated the MFL theory into the loss function and proposed a physics-informed doubly fed cross-residual network that estimated the defect length, width, and depth accurately [146].

### 5.2. Iteration-Based Defect Quantification

**Figure 14:**
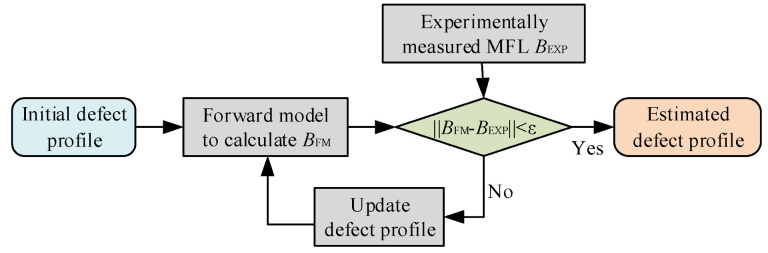
The iterative process for defect quantification in MFL testing.

Prior to the application of machine learning-based methods, iteration-based methods have been widely used in defect quantification [147,148,149,150,151,152,153,154,155,156,157,158,159]. The basic concept of iteration methods is shown in Figure 14, where the defect quantification is regarded as an optimization problem that minimizes the difference between the experimentally measured MFL signal and the one calculated with the estimated defect profile. To begin the defect quantification process, an initial estimate of the defect profile is required. Then, a forward MFL model is used to calculate the MFL signal generated by the defect profile. Usually, there are types of forward models that can be used, namely the magnetic dipole model, finite element model, and neural network model. After the calculation with the forward model, a comparison is made between the calculated MFL signal and the one obtained in the experiment. If the error is less than a desirable value, the profile will be regarded as the final estimation, otherwise, the error is used to update the defect profile and repeat the process of forward calculation.

Since defect quantification is regarded as an optimization problem, many optimization algorithms can be used to update the defect profiles. More conventionally, the gradient descent algorithm is used [160,161,162]. Later, genetic algorithms [162], particle swarm optimization [163,164,165], and cuckoo search [166,167] have been applied to quantify defects in MFL testing.

## 6. Applications and Comparison with Related NDT Methods

### 6.1. Applications of MFL Testing

As an efficient nondestructive testing method for ferromagnetic materials, MFL testing has been successfully applied in many industrial fields. One of the most important applications is underground pipeline inspection, where the so-called pipeline inspection gauge (PIG) is used. The PIG usually has permanent magnets as the magnetizing units, ferromagnetic yokes to connect the magnets and form a magnetic circuit, and brushes to separate the magnetizer and pipes. 

**Figure 15:**
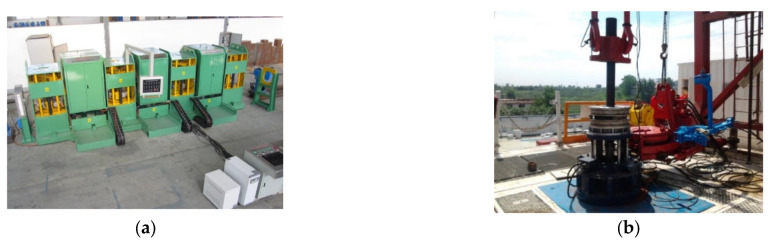

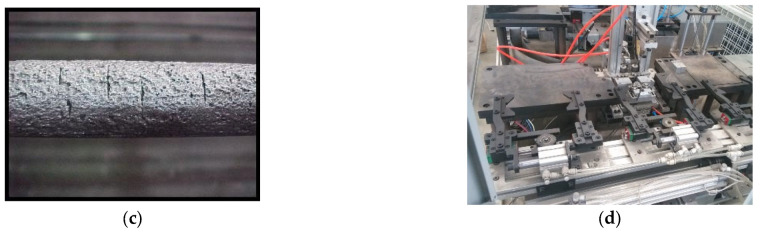
Applications of MFL: (**a**) seamless steel pipe; (**b**) drill pipe; (**c**) sucker rod; (**d**) bearing.

Another important application is the inspection of steel pipes during manufacturing. According to the API standard, the steel pipes must be tested before leaving the factory. Among the testing methods, the MFL is the most commonly used. Typical MFL equipment for seamless steel pipe is shown in Figure 15a. It consists of three modules, two of which are for the inspection of transverse and longitudinal cracks and a demagnetizing module to demagnetize the pipe after inspection. For the inspection of transverse cracks, encircling coils have been used to generate axial magnetic fields in steel pipes. For the inspection of longitudinal cracks, magnetizers with two shoes that are 180° away from each other are used to generate magnetic fields in the circumferential direction. In the oil industry, MFL has also been applied in the inspection of drill pipes and sucker rods as shown in Figure 15b,c.

In the automobile industry, bearings were previously tested by the method of magnetic particle inspection (MPI). MPI has good sensitivity for tiny cracks; however, the inspection result is dependent on the analysis of the inspector. With the usage of highly sensitive sensors, MFL can also achieve the detection of tiny cracks. In addition, MFL has the advantage of automatic inspection; thus, it is replacing MPI in several fields, such as the inspection of bearing, as shown in Figure 15d.

**Figure 16:**
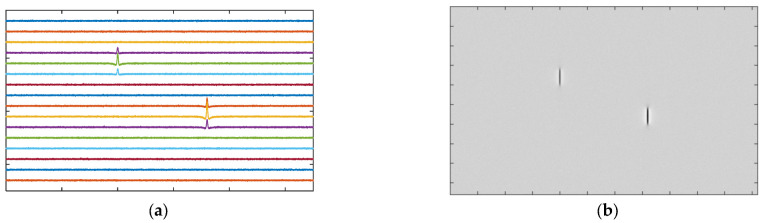
Visualizing MFL testing signals: (**a**) multi-channel time-domain signals; (**b**) gray-scale images.

In industrial applications, usually, an array of magnetic sensors is used to cover the whole area of the specimen. There are two common ways to display and visualize multi-channel signals. The most typical way is to display the signals one by one in the time domain as shown in Figure 16a. With the rapid development of image processing technology, especially deep learning techniques such as CNN, displaying the MFL testing results as gray-scale images (Figure 16b) would facilitate the application of corresponding algorithms to extract defect information.

### 6.2. Comparison with Related NDT Methods

MFL technique belongs to the category of electromagnetic NDT. Within this category, there are other methods such as eddy current testing (ECT), magnetic particle inspection (MPI), metal memory method (MMM), magnetic Barkhausen noise (MBN) method, permanent perturbation (PMP) method, magnetic adaptive testing (MAT) method, and magnetic permeability perturbation (MPP) method. The advantage of MFL over MPI is the easiness of implementing automatic testing; however, at the same time, it has lower sensitivity than MPI. When compared with ECT, MFL has better detection ability for deeply buried defects, whereas it has lower sensitivity for surface defects.

The comparison between MFL and newly proposed electromagnetic NDT methods has been discussed in some previous publications. G. Vértesy applied MFL, MMM, and MAT to detect an artificial slot in stacked steel plates, he found that the MFL gave good results when there are one or two layers and MAT outperformed MFL when there were more layers [168]. Z. Deng compared MFL with MPP and found that MPP can detect defects that are buried deeper than MFL [169]. Y. Sun compared MFL with PMP and stated that the PMP method can accomplish inspection in a narrower operation space and is more suitable for omni-directional cracks [170,171].

## 7. Conclusions

In this paper, a comprehensive review of the MFL technology has been presented. Firstly, the principle of MFL testing has been explained with the theory of refracted magnetic field and an analytical expression for the leakage magnetic field has been derived based on the 3D magnetic dipole model. Then, the influence of some crucial factors, such as defect size, defect orientation, liftoff distance, magnetization strength, testing speed, surface roughness, and stress, on the MFL testing signal has been analyzed.

Excitation and sensing are the most important steps in MFL testing, in which excitation decides if there is a leakage field generated, and sensing decides if the generated field can be effectively detected. In this paper, the development of magnetizer structures and the usage of different excitation signal waveforms have been introduced.

In the quantification of defects, there are mainly two types of algorithms, namely the machine learning-based algorithm and the iteration-based algorithm. Both algorithms achieved relatively good accuracy on defect quantification. The machine learning-based algorithm usually requires a large training set, and the iteration-based algorithm usually requires large computational resources during the iteration process.

With the advantages of high efficiency, low cost, and environmental friendliness, MFL has been applied in many applications such as underground pipelines, seamless steel piles, drill pipes, sucker rods, and bearings.

## Data Availability

Not applicable.
